# “Lack” and “Finally”: A Qualitative Analysis of Barriers and Facilitators in Rare Disease Healthcare

**DOI:** 10.3390/ijerph22010117

**Published:** 2025-01-16

**Authors:** Amanda R. Hemmesch, Kathleen R. Bogart, Erica Barnes

**Affiliations:** 1 Department of Psychology, St. Cloud State University, St. Cloud, MN 56301, USA; arhemmesch@stcloudstate.edu; 2 Department of Psychological Science, Oregon State University, Corvallis, OR 97331, USA; 3 Minnesota Rare Disease Advisory Council, St. Paul, MN 55104, USA; erica.barnes@state.mn.us

**Keywords:** rare disease, healthcare access, barriers, facilitators, COVID-19

## Abstract

**Background:** This study explored the experiences of adults with diverse rare diseases (RDs) and RD caregivers with barriers and facilitators to healthcare access in the United States (US), including during the early part of the COVID-19 pandemic, and their recommendations for improving access. **Results:** Adults with RDs and parents/caregivers to children with RDs (N = 1128) completed open-ended survey items. Responses were analyzed using thematic analysis. The primary theme identified regarding barriers to healthcare was “lack”; participants reported challenges in obtaining an accurate diagnosis, effective management/treatment, health insurance coverage, and social support. The primary theme identified regarding facilitators was “finally”; participants reported a need for persistence to access a diagnosis, RD experts, as well as social support and advocacy. Recommendations for improving healthcare for RDs mirrored the barriers and facilitators identified, including improving knowledge/awareness of RDs and investing in RD research that could improve diagnosis and treatment. Participants’ healthcare experiences varied widely during the COVID-19 pandemic, with some reporting that telehealth improved care and others reporting disruption due to telehealth. **Conclusions:** Even though individual diagnoses are rare, there are shared challenges to healthcare access and common opportunities for improvement. Policy recommendations regarding RD healthcare focus on improving affordable and timely access to knowledgeable providers, diagnosis, and medications/treatments.

## 1. Introduction

Even though any individual rare disease affects fewer than 200,000 people in the US, approximately 10% of the population has a rare disease (RD; [1]). Research suggests that some experiences may be shared across rare diagnoses. For example, previous research on RDs from around the world has identified challenges to accessing healthcare for RDs, including difficulties finding general practitioners and specialists who are knowledgeable about rare conditions [2,3], lack of information about medications and treatments [4] or confusing information about conditions and treatments [2], and diagnostic odysseys that include multiple providers and delays [5]. Bogart, Hemmesch, and colleagues conducted a large, recent study of RDs in the United States that shows similar issues for adults and parents/caregivers navigating US healthcare systems [6]. Approximately one third of that sample waited over four years for a correct diagnosis and a majority of participants reported seeing two or more providers before receiving a diagnosis, with many participants rating their initial providers as having poor knowledge of RDs. This study also found that participants reported difficulties with health insurance, including delays and denials for diagnostic tests, medications, and treatments, and referrals to out-of-network specialists. The present study aimed to provide a deeper understanding of barriers to healthcare access.

## 2. Literature Review

Challenges navigating healthcare systems may contribute to poorer quality of life for people with RDs. Bogart and colleagues [6,7] found that adults and children with RDs showed poorer health-related quality of life than the general US population and Americans with more common chronic conditions. Bogart, Hemmesch, and colleagues also showed that participants generally rated their satisfaction toward their healthcare providers as neutral; feeling less stigma, anxiety, pain, sleep disturbance, and diagnostic delay, and more physical functioning were associated with greater satisfaction with providers [6].

Another large content analysis study of RDs in the United States examined challenges reported by adults living with RDs [3]. Some of these challenges were related to healthcare access, including issues with insurance coverage and financial distress related to medical expenses or difficulties working. Other challenges were related to living with RDs more generally, such as a lack of instrumental and emotional support, distress, stigma, guilt, and issues with work and education. Including open-ended questions in this study helped to broaden understanding of the lived experiences of adults with RDs in the US, especially considering that much RD research is narrowly focused on symptoms and treatments. Other work demonstrated that psychosocial issues related to living with RDs, specifically stress and emotional and companionship support, are associated with satisfaction with life [8].

Themes of “a shock to the (health) system”, coping with uncertainty, and the value of social support while isolated were identified in a qualitative study of the psychosocial experiences of 759 adults with RDs during the first wave of COVID-19 [9]. Challenges included psychological distress, a lack of regular social and informational support, limitations in healthcare access, and subsequent exacerbation of symptoms. Silver linings were also identified, including increased access to telehealth, and the potential for the pandemic to spur structural change to address social and health inequities that people with RDs face.

Much of the previous large-scale research about the impact of RDs has focused on measures of death/disability, medical costs, and financial/productivity costs [10,11]. Bogart and colleagues [6,7] found that adults and children with RDs showed poorer health-related quality of life than the general US population and Americans with more common chronic conditions. Research outlining individual-level experiences navigating RDs has suggested unique challenges to living with RDs though few studies have recruited samples representing numerous diagnoses. The aim of the current study was to build on previous research by including open-ended questions in a large survey of adults with RDs and parents/caregivers to children with RDs. Because of the dearth of qualitative research addressing commonalities across RDs, the goal of this project was to explore participants’ experiences navigating US healthcare systems in their own words. Specifically, we aimed to learn more about barriers and facilitators to healthcare access and how the COVID-19 pandemic may have influenced healthcare access for individuals with RDs in the US.

## 3. Materials and Methods

### 3.1. Participants

We recruited adults living with one or more RDs or parents/caregivers to someone living with at least one RD for this study. Participants who recovered or died from their RD were excluded to maintain a focus on current lived experience with RDs. Participants were recruited using multiple strategies, including flyers shared via hospitals and healthcare providers, mailing lists and social media posts from RD support organizations, and snowball sampling. A total of 1639 participants responded to this online survey. Participants who did not click “yes” to consent to participation, were under age 18, recovered or died from their RD, or did not self-report at least one RD were removed from the dataset prior to analysis. When multiple entries existed from the same IP address, the earliest or most complete submission was retained for analyses and others were removed. The final dataset included 1128 participants with verified RDs.

### 3.2. Measures

This survey included both quantitative and qualitative questions. The focus of this paper is responses to qualitative questions about healthcare access to provide additional depth and detail to provide context for the quantitative findings. Qualitative questions were also included to give participants an opportunity to identify experiences with RDs that may not have been captured by the quantitative surveys used in previous research. The qualitative questions were developed in consultation with the Minnesota Rare Disease Advisory Council’s Barriers to Care Working Group, which included representatives from a variety of healthcare fields (e.g., medical doctor, dentist, pharmacist). Quantitative results are available elsewhere [6].

***Barriers and Facilitators to Healthcare Access*** Participants were asked to share their three most significant barriers and facilitators to diagnosis, management, or treatment of their RD. Barriers were designed to capture experiences that have felt difficult or frustrating; facilitators were designed to capture experiences that felt positive or helpful. Approximately 950 participants responded to the item about barriers and 900 responded to the item about facilitators.

***One Single Item to Improve Healthcare Access*** Participants answered an open-ended question about what one thing they thought would most improve their healthcare experience. Approximately 750 participants responded to this question.

***Changes to Healthcare Access during the COVID-19 Pandemic*** The survey included a question asking participants if the COVID-19 pandemic changed their access to healthcare. If participants indicated “yes”, they had the opportunity to elaborate upon that answer with an open-ended response. Approximately 450 participants responded to this question.

***Participants’ Final Thoughts/Anything Else to Add*** The last item of the survey asked if participants had anything else to add. Approximately 500 participants responded to this final question.

### 3.3. Procedure

Qualtrics, an online survey platform, was used to collect data for this study, though participants could request a paper copy of the survey be mailed to them if they preferred to participate that way. Participants were presented with informed consent information on the first page of the survey. Implied consent was recorded if participants clicked “yes” that they understood the nature, risks, and benefits of the survey. Data collection occurred from October 2020 through February 2021, during the COVID-19 pandemic. The survey took approximately 30 min to complete and participants were not compensated for their time.

### 3.4. Data Analysis

We used a thematic analysis approach for qualitative data analysis [12]. Thematic analysis is relatively flexible and can be used to identify themes in qualitative data whether or not existing theories can be used as a lens for interpreting data. This approach was selected because it balances flexibility with clearly defined methods [12]. Themes identify a pattern within the data that “captures something important” about participant responses to the topic [12]. The goal is to identify themes that capture the richness (in terms of breadth or depth) of the data in relation to the research questions, not to quantify those themes. Because little theory exists that identifies potential barriers or facilitators for healthcare access in RD, we used a multi-step, inductive approach to data analysis.

For each open-ended item, a team of three or four coders (two or three undergraduate research assistants and the first author) completed the following steps: data familiarization, generating initial codes, searching for themes, reviewing themes, and defining themes. Due to changing availability, additional undergraduate coders were recruited to replace those who were no longer able to participate in data analysis for this project. During data familiarization, coders independently read participants’ responses at least twice. When generating initial themes, coders took notes about items that seemed to express something important about responses to each item. After each coder identified their own initial themes, coders met weekly to discuss their notes and identify themes that were agreed to represent important information in the dataset. Coders decided that this data included both overarching themes and more specific subthemes, or types, of barriers and facilitators. All of the themes and subthemes/types described in this manuscript were finalized when coders achieved consensus (i.e., all coders agreed on the theme and its description). Within the parameters of thematic analysis, agreement measures such as inter-rater reliability are not appropriate [13]. The coding team did not examine potential differences between people living with RD or caregivers because the goal of this analysis was to identify common, shared experiences. Readers interested in group comparisons are encouraged to read Bogart et al. [6].

## 4. Results

### 4.1. Sample Characteristics

The majority of participants were adults with RDs (70%), women (80%), and white (90%). Participants represented 344 different rare diagnoses; most participants reported one RD (88%), though some participants reported as many as five RDs. See Bogart et al. (2022) for additional information about this sample [6].

### 4.2. Barriers and Facilitators to Healthcare Access

Coders identified two overarching themes that captured participants’ responses to the questions about their three biggest barriers and facilitators to healthcare access, as well as ten types of barriers/facilitators. Coders identified similar subthemes/types for the item about “one single thing to improve healthcare access” to those identified for barriers and facilitators, so results of those subthemes/types are presented together. Overarching themes are presented in this section, while a detailed list of types of barriers and facilitators/improvements, including descriptions and example quotations from participants, can be seen in Table 1.

### 4.3. Barriers Theme: “Lack”

The primary theme that was identified when reviewing participants’ responses about barriers to successful diagnosis, treatment, or management of their RD was “lack”. A sense of lack or absence was pervasive across the types of barriers identified. For example, many participants wrote about how the absence of a doctor/healthcare provider knowledgeable in RD hindered their ability to obtain an accurate diagnosis and pursue treatments to manage symptoms. For example, “doctors (specialists and PCP’s)… were disinterested in making an effort beyond the 20 min appointment to diagnose me” (woman in her 60s with conversion disorder). Many participants also wrote about the lack of a cure for their RDs, and sometimes a lack of (effective) treatments to manage symptoms. A lack of knowledge and awareness about their specific RD and RDs in general was also identified as a barrier. Participants wanted more to help them manage their RD, including more research about diagnosis and treatments for RDs. Participants wrote about the need to persist and to advocate for themselves as they tried to manage their RD. “Lack” was associated with a variety of negative participant responses, including frustration, anxiety, depression, stress, and stigma.

### 4.4. Facilitators Theme: “Finally”

The primary theme identified when participants considered facilitators was “finally”. Participants wrote about the persistence needed to manage their RD and to navigate healthcare systems. For example, many participants who identified doctors and other healthcare providers as facilitators wrote about how they had to search to find the right doctor/provider, one who did not dismiss their concerns and who seemed knowledgeable about their RD. For example, “[The biggest facilitator was] one specialty care provider’s support and care during my journey who believed me and has done what she can to provide guidance and hope. If it wasn’t for her I don’t think I could have made it to diagnosis before giving up all hope of ever finding answers or receiving treatment” (woman in her 30s with non-radiographic axial spondyloarthritis). Participants often reported diagnostic odysseys as barriers; however, participants also highlighted the importance of an accurate diagnosis as a facilitator for managing their RDs and accessing healthcare. Relatedly, participants wrote about their experiences seeking treatments to manage their RD symptoms that felt like trial-and-error, and expressed relief when they were able to receive a medication or treatment that improved their quality of life. Participants also identified personal attributes as important in their RD experience, like optimism, persistence, and a positive outlook. As a complement to internal factors, participants highlighted the importance of social support from various sources (e.g., family, friends, and online support communities) as important for their ability to manage their RDs. Although many different facilitators were identified, many participants expressed relief upon finally finding the resources that were important to their RD experience. In addition to relief, facilitators seemed to bring feelings of validation and the opportunity for greater inclusion and improved quality of life.

### 4.5. One Thing to Improve Healthcare Access in the US

Coders identified ten types of improvements that could be addressed to improve healthcare access and RD management (see Table 1). These were the same general categories that were identified as barriers and facilitators, including changes related to training for healthcare providers/doctors, improvements in diagnostic testing, additional research and development devoted to finding medications and treatments, improved communication between patients/families and providers and among providers (including access to a common medical record systems), more research about RDs in general, changes related to health insurance coverage that reduce costs, improved access and disability accommodations, increasing knowledge, awareness, and education regarding RDs, better formal and informal social support for patients and families, and building more empowerment and advocacy skills.

An interest in care coordinators or mentors who could help participants and their families navigate the healthcare system was also identified (included as part of the subtheme pertaining to social support as a barrier and facilitator in Table 1). Many participants thought that care coordinators would be a significant improvement to current healthcare systems because they could facilitate communication with and among healthcare providers, scheduling (especially when participants needed to see multiple providers or schedule multiple tests), and provide an additional access point to healthcare systems. Care coordinators could also advocate on behalf of patients and their families to help them identify additional options for diagnosis, medication, treatment, and support to complement information from traditional medical providers.

### 4.6. Changes to Healthcare Access During the COVID-19 Pandemic

Coders identified five themes in participants’ responses about how the COVID-19 pandemic affected their healthcare access, including: lack of access, delays and availability issues, poorer quality of care, COVID-specific restrictions, and virtual appointments/telehealth. Data collection occurred during the early part of the pandemic (before widespread availability of vaccines in the US), which likely shaped responses to this question.

The COVID-19 pandemic caused a relatively sudden lack of access to a variety of healthcare options in the US, including appointments with doctors/healthcare providers, dentists, supportive therapies (e.g., gyms, physical therapy), clinical trials, and hospital, school, and in-home services. This sudden change to schedules meant that healthcare was canceled or delayed. For example, “Diagnostic tests have taken up to six months to schedule because my conditions are not life threatening. Even with my advocating for care and nagging my providers, many things have been significantly delayed” (woman in her 20s with narcolepsy). For some participants, this exacerbated processes that moved slowly before the pandemic, like diagnostic testing and scheduling appointments with specialists: “I have delayed treatment, both routine and surgery, and let my condition become more progressed than normal, rather than risk travel and exposure to COVID” (woman in her 30s with narcolepsy). Delays and availability issues caused an inability to plan for future visits or treatments, which influenced the progression of some people’s RDs. Relatedly, some medications were more difficult to get refilled during the pandemic because participants were unable to schedule appointments with healthcare providers or carry out regular testing. This was especially true for individuals who were prescribed controlled substances to manage their RD symptoms. Some participants also reported poorer quality of healthcare during the pandemic because doctors were busy treating patients with COVID-19 or were burned out, because their appointments and treatments were delayed/canceled/rushed, or ongoing RD research was canceled: “Everything is 1000x harder and takes longer” (woman in her 30s with idiopathic hypersomnia).

“…As a practitioner myself what COVID has changed the most is the elimination of a support infrastructure for all providers so that staff is now responsible for so, so many more roles. The overall bureaucracy of management is assigning more and more tasks and responsibilities without a corresponding increase in staff to complete these jobs. Burn out is coming and the healthcare system is in real jeopardy of losing the professionals that the system requires. For example within the hospital setting there are no more volunteers to complete many of the tasks that ensure patients needs are met. RNs now have an expanded role, support positions are being eliminated (birth certificates clerks to unit secretaries to CNAs) and, you guessed it, RNs can take on that as well. MDs are being spread thin by taking on a broader scope of territory (more units and/or more clinics) without a corresponding uptick in staffing. Less staff is available to support residents and their clinical rotations are lacking the skill building and education seen historically. That should be an interesting repercussion” (nonbinary person in their 40s with idiopathic hypersomnia and parent to a child with RD).

Some participants also shared changes to their healthcare experiences that were specific to COVID itself and to restrictions to slow the spread of COVID. Many participants expressed fear about contracting COVID-19 and how it might interact with their RDs. Because US healthcare shifted to address the COVID pandemic, participants were unable to access in-person healthcare services unless they were experiencing emergencies or COVID-19 symptoms. When participants were able to access in-person care, COVID-19 testing was sometimes required before appointments and procedures, which added an extra barrier to healthcare access. For example, “Most services have been delayed or canceled. Accessibility is different in that there are different and new protocols for going to the doctor…” (parent of a child with Cornelia de Lange syndrome). Participants also expressed concerns about masking during the pandemic, including experiencing stigma for wearing or not wearing masks and issues with wearing masks due to their medical conditions. Finally, restrictions enacted to slow the spread of COVID were also identified as barriers to accessing healthcare. For example, travel restrictions meant that some participants could not see providers outside of their local area. Also, visitor restrictions at healthcare facilities meant that some participants were unable to bring caregivers or other advocates with them to their appointments or procedures.

Participants had varied experiences with virtual appointments/telehealth during the pandemic. For some participants, the shift to virtual appointments was a benefit because it provided easier access to healthcare providers and was more convenient because participants did not have to travel for appointments. The shift to virtual appointments also meant that more providers were accepting virtual appointments than before the pandemic, which increased options for care (though some providers were unable to offer virtual appointments across state lines). For participants concerned with COVID-19 infection and complications, virtual visits also meant that they could access their healthcare providers without risking exposure to COVID-19: “I do not feel safe going to medical offices and have been shocked at the number of people allowed in at the same time. I have either had to forgo visits/care or opt for telehealth when available” (woman in her 40s with primary biliary cholangitis). Another participant described some of the complexities of telehealth during the pandemic, “More telehealth, which is plagued by problems, but is better than going to a hospital facility during a plague” (woman in her 30s with primary biliary cholangitis).

However, the shift to virtual appointments brought some new challenges for healthcare access. Healthcare providers were unable to carry out physical examinations or tests that are important for RD diagnosis and management. Virtual appointments also made it more difficult for providers to track changes to some participants’ RDs or detect new areas of concern. The technology required for virtual appointments was also a barrier for participants who lacked technology skills, devices, or stable high-quality internet connections. Virtual appointments also made it more difficult for some participants to focus their attention because they did not have quiet, private spaces to use for their appointments.

Some participants expressed a love/hate relationship with virtual appointments because they appreciated the convenience they afford but felt they were not productive enough to manage their conditions: “We did not attend regularly scheduled clinic due to the pandemic. At our next visit (scheduled), we did telehealth services. In a child with a lung disease, this isn’t ideal as a physical examination cannot be performed” (parent of a child with cystic fibrosis). Another participant said “It was extremely difficult to find care as a new patient because doctors were reluctant to start treating someone they’d only “seen” virtually. It was especially difficult navigating insurance and new patient paperwork because I didn’t have a printer and the public libraries (my usual source for printing) were shut down. It also made it much scarier to have to go to the pharmacy every single month for my controlled substance prescription. On the positive side, pharmacies began providing free prescription delivery on many medications, which made it easier to get other prescriptions (not for my rare disease) at least. Plus, once care was established, it is nice not to have to worry about driving to a doctor since that’s difficult with my disorder.” (woman in her 30s with narcolepsy).

### 4.7. Participants’ Final Thoughts/Anything Else to Add

Coders identified some additional themes from participants’ responses to this question that can help inform future RD research and practice. One of these themes is issues related to parenting/caregiving. Parents/caregivers wanted to be included in their loved one’s care team and to be able to contribute their experiences/observations to conversations about diagnosis and treatment/management. Parents/caregivers also reported feeling exhausted at times providing care to their child with RD and wanted more support that is dedicated to their experience as caregivers. For example, “You’re missing a ton here about how the rare disease impacts family. Issues include: parents having to change jobs or quit jobs to care for child, child’s accommodations or lack thereof with school, extended family members who don’t understand the illness/think you are faking or attention seeking, impact on siblings, feeling not understood by peers (which is already hard when you’re a teenager). There’s also the lasting impact of not being believed by medical providers that makes you reluctant to seek care for your rare illness or other illness because you fear they are not going to believe you. Then there’s debt and poverty, the joys of trying to apply for disability, etc. There are layers and layers of stress.” (parent of a child with mast cell activation syndrome).

Another theme that was identified was uncertainty. Adults with RD and parents/caregivers to children with RD were often uncertain about what their RD would mean for their life, education, and work, and wanted a road map to help navigate multiple systems with their RD experience. The COVID-19 pandemic added to this feeling of uncertainty. Participants wanted others to understand this as a separate part of their experiences with RD.

An additional theme captured the socioemotional challenges of having a RD. These included feelings of anxiety, depression, stress, isolation and loneliness, and feeling misunderstood and like they needed to convince others that their diagnosis was valid. More patient and family support is needed to help people with RD and their families manage these emotional challenges. A few participants expressed a desire for more support for medical assistance in dying for people with untreatable and intractable RDs. Some participants shared that they felt they obtained better information and support from the internet and RD Facebook groups than from healthcare providers. Participants expressed interest in participating in research and clinical trials to help improve diagnosis and management of RDs so they could potentially improve quality of life for others with their RD.

Finally, participants expressed gratitude and appreciation. Participants were thankful for diagnosis because that helped them to obtain access to medical support and resources that could improve their quality of life. Participants expressed gratitude for financial stability or financial support like waivers because these helped make costly appointments and treatments attainable for RD management. Finally, participants expressed gratitude for knowledgeable providers and research being conducted on RDs, especially because there is so much unknown about RDs that contributes to challenges mentioned above.

## 5. Discussion

This study built upon previous research by using a qualitative approach to assess both barriers and facilitators to healthcare access for adults and parents/caregivers to children with RD in the US. We found that adults with RDs and parents/caregivers to children with RDs experience numerous types of barriers and facilitators to healthcare access that often overlapped. For example, doctors/healthcare providers could be both barriers to quality care as well as facilitators. “Lack” was the primary theme identified when considering barriers to healthcare access—many participants felt that a lack of knowledgeable providers, medications and treatments, communication, RD knowledge, and social support made it difficult for them to access quality healthcare, receive an accurate diagnosis, and manage their RD symptoms. The COVID-19 pandemic exacerbated many of these barriers, providing additional challenges for navigating US healthcare systems. “Finally” was the primary theme identified regarding facilitators—many participants expressed a need to persist to find the right providers, medications and treatments, and knowledge to manage their RD symptoms. Many participants eventually were able to find the support they sought, though the process sometimes mirrored the diagnostic “odyssey” identified in other RD studies [5,10]. The pandemic complicated access to facilitators like allied health services and healthcare providers, though virtual appointments provided much needed access and flexibility for participants whose conditions could be managed from afar.

These findings replicate and extend Bryson and colleagues’ [3] content analysis study of adults with RDs, which identified challenges of living with RD such as symptoms and corresponding activity limitations, treatments, uncertainty, poor communication between providers and patients, insurance/costs, and lack of different types of social support. Both adults with RDs and parents/caregivers of children with RDs in the current study reported similar barriers when considering healthcare access, which illustrates that people with RDs and their families may encounter these challenges in multiple domains of their lives. Similar barriers have also been identified in other samples, such as the lack of providers with knowledge about mitochondrial disorders [2], diagnostic delays for various RDs [5], and lack of treatments and diagnostic tests available to people with sarcomas [4]. Direct medical costs and indirect costs such as productivity loss due to caregiving or symptoms were commonly cited by participants in studies as barriers or challenges related to RDs. These individual reports are reflective of other research that suggested that the economic impact of RDs (relative to the general population) in the US in 2019 was approximately USD 997 billion, including USD 38 billion for RD treatment/management not covered by health insurance [8], which illustrates the aggregate impact of these personal barriers.

Emphasis on social support as a facilitator is consistent with the finding that emotional support and companionship are related to life satisfaction for adults with RDs [7]. The current study suggests that social support may also facilitate healthcare access and RD management. Although social support was found to be an important facilitator, people with RDs experienced significant disruption to their support during the pandemic [12].

Stigma, feelings of uncertainty, and other socioemotional responses to RDs were present in this sample, which is also consistent with previous research [3]. Psychosocial factors like stigma, bias, and discrimination were identified as barriers to healthcare access, whereas empowerment and self-advocacy were identified as facilitators. These factors affected both adults and parents/caregivers to children with RDs. Some participants shared that they were able to persist through these psychosocial barriers, often because they perceived support from others in their lives that contributed to their resilience navigating difficult healthcare systems.

Caputo [14] identified need for autonomy as an important component of illness stories for adults with RDs, which included concern for being seen as a whole person with socioemotional needs. Three latent factors contributed to the themes identified by Caputo: relationships with healthcare providers that balance physical and socioemotional concerns, adjusting to RD limitations, and personal beliefs and coping strategies that vacillated between hopelessness and proactively shaping their lives and future. The current study identified similar themes related to RD diagnosis and management, especially the importance of doctors/healthcare providers who worked collaboratively with their RD patients during diagnosis and management/treatment. The current study—and previous research—indicate that RD treatment and management interventions should also address socioemotional needs and building resilience.

Parents and caregivers to children with RDs expressed that they wanted additional support targeted toward their experience. This complements a narrative review study that found reduced quality of life for parents of children with RDs, particularly for psychosocial outcomes [15], and a study that found that patients with mitochondrial disease identified additional social support as a clinical priority [2]. These findings suggest that more should be done to support individuals with RDs and their families as whole people with different roles in managing RDs.

This study included questions about how the COVID-19 pandemic affected healthcare access and RD management. The influence of the pandemic varied widely across participants: some found that the shift to virtual healthcare made it easier for them to manage their RD, whereas others found that the pandemic limited or restricted their access to providers and services that were important for RD management. One takeaway from this study was the desire to keep and expand flexibility in healthcare delivery options, a future outcome participants in Bogart and colleagues [12] hoped for as well. Greater flexibility (through telehealth options) provided access to people who may have struggled with access before, especially those who need to travel long distances for specialist care [6]. Telehealth options should continue to be available after the pandemic.

Similar to what Karaa and colleagues found in their patient survey and focus group, many participants in this study felt that they were responsible for coordinating care across different healthcare providers and systems [2]. As a result, many participants suggested that care coordinators or advocates would be an area for improvement in navigating US healthcare systems. Care coordinators/advocates could provide both institutional support for participants navigating and coordinating insurance, providers, and healthcare systems, as well as social support for the individual with a RD and/or their family.

## 6. Limitations

This sample included participants with a variety of rare diagnoses, as well as parents and caregivers to people with RDs. However, the sample for this study was limited to participants in the US, and was mostly white, female, and had higher socioeconomic status than the general US population, which makes them unlikely to represent the larger population of adults with RDs and parents/caregivers to children with RDs. The over-representation of white, female, and higher socioeconomic status participants is a common issue in RD research due to these demographics being most likely to connect to support organizations [16,17]. However, this sample represented over three hundred different rare diagnoses, making it one of the larger RD studies completed to date. It is likely that minoritized populations may experience these and other barriers to healthcare access to a greater degree. Future studies should recruit more diverse samples to better represent the population of adults and families navigating RD healthcare access.

Data collection for this study occurred during the early part of the COVID-19 pandemic (October 2020 to February 2021). Most questions asked participants to think about their experiences accessing healthcare in the US before the pandemic, but experiences during the pandemic may have influenced how participants responded to questions about barriers and facilitators for healthcare access. However, results are consistent with RD research published before the pandemic, which lends convergent validity to these findings and illustrates that many barriers and facilitators for healthcare access are likely to be encountered beyond the COVID-19 pandemic. Indeed, participants shared how the pandemic changed their access to healthcare for both better and worse.

## 7. Conclusions

The current student identified common types of barriers and facilitators to healthcare access in the US across RDs. The overarching barrier identified was a sense of “lack” that pervaded multiple domains of healthcare access and RD management. The overarching facilitator identified was “finally”, which captures the persistence required for healthcare access for RD diagnosis and management in the US. Doctors/healthcare providers, diagnosis, medications/treatments, communication, research, insurance, accessibility, knowledge/education, social support, and psychosocial factors like stigma and self-advocacy were experienced as both barriers and facilitators. Patient and parent/caregiver recommendations for improving healthcare access were similar to the types of facilitators people experienced, and included establishing or expanding care coordinators to help address numerous potential barriers. Additionally, adults with RDs and parents/caregivers to children with RDs suggested that more training for healthcare providers about RDs and how to communicate well with patients/families, removing barriers to diagnosis, continued research about RDs to better understand etiologies and treatments, and changes to US health insurance could improve healthcare access in the US. Psychosocial factors emphasize the need for systems to treat individuals with RDs and their families in a holistic manner because they are related to healthcare access and successful RD management. The impact of the early part of the COVID-19 pandemic varied widely for adults and families managing RDs, as did participants’ experiences with the expansion of telehealth options. These findings are consistent with RD research from other Western samples studied before the COVID-19 pandemic, and emphasize the need for building awareness and infrastructure across specific rare diagnoses to meet the needs of the broader RD community.

## Figures and Tables

**Table 1:** Types of barriers and facilitators to healthcare access for people with rare diseases and their parents/caregivers.

Type	Description	Examples
**Healthcare providers/doctors**	**Barrier** Participants shared that they felt healthcare providers did not listen to them or were dismissive (sometimes attributing RD issues to mental health conditions), did not care enough about them or finding a successful treatment, did know know enough about RDs, were not able to see beyond their own specialties to understand their patients as whole people, and communicated poorly. **Facilitator/Improvement** Participants appreciated when healthcare providers listened to them and believed them/treated them as reliable reporters, communicated complex medical information in accessible language, were compassionate, and had some knowledge of their RD. Participants recommended that healthcare providers do more to address biases in the medical system related to race, gender, socioeconomic status, and weight that can compromise quality of care.	**Barrier** “Doctors will simply do anything they can to invalidate the severity of my concerns when I have, at this point, been living in my body for almost a quarter of a century. I am all too aware of what is normal and what is not, and yet no one wants to listen when I can explain things like “I’m in so much pain I can’t sleep at night. It wakes me up every few hours and it’s agonizing.” Doctors will argue with me about what I need to do even though I explain that I’ve ALREADY tried what they’re suggesting. They just won’t listen.” (woman in her 20s with idiopathic hypersomnia) **Facilitator/Improvement** “New doctor acknowledging the usual nature of my constant state of tiredness and relating symptoms, actually working with trying to improve my situation and prescribing medication instead of instantly assuming it was anxiety when mentioned. He also explained to my family how it feels to have the disorder to help understanding.” (woman in her 20s with idiopathic hypersomnia) “Compassion and listening. We have had wonderful doctors/practitioners over the years and many that didn’t make the mark. Those latter individuals did not take the time to listen to us and/or made judgements prior to seeing us (“I don’t have experience with this syndrome, so we should send you elsewhere”). With this ultra-rare syndrome, NO ONE has experience. We just need doctors to listen, be willing to collaborate and find out where we need to go. AND, they have to be allowed to do that and not be rushed with time constraints.” (woman in her 60s with primary biliary cholangitis)
**Diagnosis**	**Barrier** Not having an official diagnosis, misdiagnosis, and long wait times to obtain diagnosis were identified as barriers to healthcare access. Participants shared diagnostic odysseys involving multiple practitioners and specialists before receiving accurate diagnoses that opened the door for treatment and management of their RDs. **Facilitator/Improvement ** Having a correct diagnosis was important for accessing treatments (when available) and for validating participants’ experiences with their RDs.	**Barrier** “After onset of symptoms, and a sense of worsening with time, it became discouraging and frustrating going to provider after provider and many not able to diagnose due to normal test results, or referred me to other specialists due to the nature of my symptoms” (woman in her 50s with tracheobronchomalacia) **Facilitator/Improvement** “The most helpful support was getting the right diagnosis. And although there is no cure, the treatment plan literally gave me my life back.” (473; woman in her 30s with idiopathic hypersomnia) “With an accurate diagnosis, I am able to anticipate certain changes in my physical condition and hence, make necessary decisions related to those anticipated changes.” (woman in her 50s with inclusion body myopathy with early-onset Paget disease) “I just wanted to be heard. Before my diagnosis I felt like I was talking into a void. I remember my GP looking at my blood pressure and declaring that I have the blood pressure of an athlete. I remember saying, but I’m not an athlete. I’m dizzy all the time. I had so many of these exchanges, year after year after year. I remember being so happy when my liver enzymes were too high on a metabolic test because I thought, “Now she has to listen to me. Now get tested and they’ll figure out what’s wrong with me.” They did a liver biopsy, found nothing wrong, told me that I had an unknown variety of hepatitis and I would probably feel better in a couple months, and that was it. That’s the only treatment and test I ever got and I never felt better after a couple of months.” (woman in her 20s with idiopathic hypersomnia)
**Medications and treatments**	**Barrier** RD symptoms were identified as barriers to healthcare access, especially those related to mobility, communication, fatigue, sensory, and neurological issues. Participants shared that effective medications or treatments were lacking for their RDs, which was a barrier for management. As a result, they felt they had few options for managing their RD symptoms. Pharmacy issues also created barriers for accessing RD medications. **Facilitator/Improvement ** Some participants were able to find treatments to help them manage their RDs. The treatment options available included medications (e.g., traditional, off-label, and experimental), medical services (e.g., occupational, physical, or speech therapy), and lifestyle modifications (e.g., dietary changes, exercise). When considering how to improve RD treatment and management, some participants wanted greater acceptance/exploration of holistic and alternative therapies (e.g., naturopathy, chiropractic care).	**Barrier** “I am currently in a situation where the insurance company will not approve any medication to treat my condition because there is no FDA approved medication for Idiopathic Hypersomnia.” (woman in her 50s with idiopathic hypersomnia) **Facilitator/Improvement** “I finally found a wonderful neurologist who understood my problem, had helpful suggestions, prescribed necessary medications, and was super supportive. He was my doctor for about 20 years. He retired last year. And now the fight continues with the new doctor about what medications I need.” (nonbinary person in their 60s with cervical dystonia) “I think there would be a lot of benefit for other holistic/alternative treatments to be considered, not just prescription medication.” (woman in her 60s with spinocerebellar ataxia) “Better treatment options. There are no medications approved specifically for my condition. All options are prescribed off-label and only address the symptoms, they don’t treat the underlying cause.” (woman in her 40s with hyperacusis)
**Communication**	**Barrier** A lack of communication between patients/families and providers and among providers made it more difficult for participants to coordinate their care. Parents/caregivers shared issues with accessing their dependent’s health care information or not being able to attend appointments. **Facilitator/Improvement** Clear communication between providers and participants/their families and between providers was identified as an important component of successful RD diagnosis and management. Participants appreciated having multiple modalities for communication, including electronic medical records and online messaging with their providers, that made it easier to reach providers.	**Barrier** “Multiple systems are involved, requiring multiple specialists. Communication among them is difficult.” (parent of a child with Takenouchi–Kosaki syndrome) “Finding a doctor who will listen to me” (woman in her 40s with spinocerebellar ataxia) **Facilitator/Improvement** “I love the patient portals that my doctors recently instituted, so I can leave messages late at night and receive them when it is convenient without having the bother of answering a ringing phone. Text messages from my cell phone are also wonderful.” (woman in her 70s with spinocerebellar ataxia) “Developing a care team that communicates with each other. Primary care physician, movement disorder specialist, ER doctors, PT, OT and mental health therapists communicate with each other and have shared access to my records” (woman in her 60s with multiple system atrophy) “A better understanding that it takes a team of doctors to treat and they all need to communicate on the care plan going forward. A better line of communication between hospital systems in emergencies… Also an understanding from the medical community that they need to look to other research facilities that may have more up to date experience treating these diseases instead of waiting for years for standards of care to be updated.” (woman in her 70s with spinocerebellar ataxia)
**Research**	**Barrier** Participants reported that a lack of research on RDs contributed to a dearth of diagnostic tests and treatment options for RDs. **Facilitator/Improvement ** Participants wanted more research and development regarding diagnosis and treatment of RDs, including clinical trials. They also wanted more information about participating in research opportunities for their RD.	**Barrier** “When is a disorder nobody knows in the US, nobody bothers to research it. I have to do my own research and guide my providers on meds and tests, it’s very frustrating. There is no research being done locally either so I can’t participate in anything with people that specialize. Even the geneticist kaiser sent me to didn’t know my disorder…” (woman in her 40s with familial Mediterranean fever) **Facilitator/Improvement** “Having access to continued care—taking part in research—being followed to help others in the future.” (woman in her 50s with hypophosphatasia, renal glycosuria, and Fanconi anemia) “Data feed of new research, tips from other patients, links to resources & workarounds. NOT a “patient support forum” or newsletter. I need techniques & solutions, not Hallmark cards.” (woman in her 30s with narcolepsy)
**Insurance and costs**	**Barrier** Costs and financial issues were commonly reported as barriers for healthcare access. Insurance denials, delays, waivers, and a lack of insurance were also commonly reported. High costs for diagnostic tests—especially genetic testing—made it difficult to obtain a correct diagnosis. High costs for medications and treatments—and issues with insurance coverage—made it difficult for some participants to manage their RD well. High costs related to seeking care from out-of-network healthcare providers and travel to specialists were also a barrier. In addition to financial costs, many participants identified time as a cost/barrier as they sought diagnosis and treatment/management. Canceled and delayed appointments were a cost that emerged related to the COVID-19 pandemic. **Facilitator/Improvement ** Numerous areas for improvement were identified related to cost, including addressing insurance issues such as the need for prior authorization, delays and denials, provider network limitations, as well as improving coverage for genetic testing, pharmacological treatments, off-label and experimental treatments, holistic and alternative treatments (especially when no other treatments are available), and access to specialists. Some participants also recommended improving coverage and pay for in-home and respite care.	**Barrier** “MOST significant barrier has been the management of insurance and associated co-pay programs for medications—specifically—if our social worker had not told us about the different programs, we would have no idea they exist and would be pay THOUSANDS of dollars in medicine alone.” (parent of a child with cystic fibrosis) **Facilitator/Improvement** “I have been fortunate in having excellent health insurance through the state of Minnesota so having cystic fibrosis diagnosed as an adult has not been a financial burden for us despite the fact that my medications cost close to $400,000 per year.” (man in his 70s with cystic fibrosis) “Remove the insurance/specialty pharmacy/pharmacy benefit managers/orphan drug/pharma bro corruption and red tape. My child’s specialty medication has list price of $500,000 per year, and the thought of losing my current health insurance plan keeps me up at night to the point that we have explored moving to Canada.” (woman in her 20s with Duane syndrome)
**Accessibility and accommodations**	**Barrier** Many participants traveled over 60 miles for their care, which created accessibility issues due to the lack of local care options. Some participants also shared that they experienced issues with accessible disability parking and a lack of understanding regarding their mobility or sensory aids when accessing their healthcare providers and at work and school. **Facilitator/Improvement ** Participants appreciated when healthcare facilities were proactive about mobility and sensory accommodations, including disability parking. Participants recommended either more locations for specialist care or more flexibility for accessing care (e.g., telehealth or video visits across state lines). Participants also felt that their care would be more accessible if they would have shorter waits to see healthcare providers, easier referrals, and could schedule their appointments to be on the same day at the same place.	**Barrier** “Unable to obtain quality specialized care in home area, must travel to specialty centers 4 h away” (woman in her 40s with hyperacusis and misophonia) “[Lack of] Helpful accommodations due to the nature of symptoms.” (woman in her 20s with idiopathic hypersomnia) **Facilitator/Improvement** “Any kind of accessibility helps out. From elevators to offering telehealth appointments so I don’t have to come into an office. Anything like that helps a lot.” (woman in her 20s with idiopathic hypersomnia) “Sleep neurologist suggesting and helping me submit an Accommodation Request form to my employer. I didn’t know that was available and it helped me keep my job and sanity.” (woman in her 20s with idiopathic hypersomnia) “I feel telemedical should be reviewed more thoroughly especially since many specialist of rare diseases are located across the USA. The regulations that restricting a doctor from seeing patients in any state needs to be addressed the determine how to make it more convenient for the patient and still comply with new regulations.” (woman in her 40s with eosinophilic gastroenteritis)
**Social support**	**Barrier** A lack of acceptance and social support were reported, along with feeling isolated and rejected. **Facilitator/Improvement ** Social support from multiple sources was identified as a facilitator. This included informal support from family, friends, spouses/partners, communities, and online RD sources like peer support groups, Facebook groups, and websites. Formal support sources included local, national, and international RD support/advocacy groups, conferences, support groups, and healthcare providers. Regarding improvements, participants wanted more support, especially around diagnosis (for both patients and their families) and opportunities to meet others with their diagnosis or to meet people with RDs in general. Care coordinators or RD mentors were highlighted as potentially being able to provide social support and navigate healthcare systems.	**Barrier** “When there’s not a straightforward answer or easily identified disorder either they don’t believe you or they don’t know what to do with you or where to send you so you’re often pretty much left on your own to keep trying different people have random till you find someone who can help” (woman in her late teens with autoimmune encephalitis and parent to a child with RD) **Facilitator/Improvement** “General sympathy from family, friends, co-workers. It’s important for them to know I’m not just a lazy person, that there is a reason why I have difficulty working or meeting sometimes, or that I sleep so much. The fact that I can point to a diagnosis validates my symptoms to the people around me.” (woman in her 20s with idiopathic hypersomnia) “My son says that I am his most significant facilitator. I have been there supporting hing him, holding his hand, encouraging & protecting him the whole way thru this. I am also a fierce advocate for his care and I research constantly trying to find anything that might help him. I would add that equally significant is Dr. NAME…” (relative/caregiver to a child with transient global anemia) “I would say that the most helpful thing I have encountered is the [Recurrent Respiratory Papillomatosis Foundation] support group on Facebook. The people on there are knowledgeable, friendly and encouraging. Any time I have had a question, they have answered it and when I have been most discouraged, they have cheered me on…” (woman in her 50s with recurrent respiratory papillomatosis) “Care coordination and support. Like a social worker or nurse case manager to help me manage everything including medical doctors, medications, and school, education, therapies, IEPs… It is very difficult to manage it all.” (woman in her 20s with antiphospholipid syndrome)
**Knowledge, understanding, and education**	**Barrier** Participants identified a lack of knowledge and education as a barrier to care at many levels, including personal challenges finding and understanding information about their RD, as well as providers who were not familiar with their RD and were not interested/able to carry out additional research about potential diagnoses and treatments. Participants also identified a lack of awareness about RDs generally as a barrier. **Facilitator/Improvement ** Participants felt empowered to learn more about their RDs themselves. More RD education for providers, perhaps even a universal database for symptoms that would be accessible across healthcare systems, was identified as an area for improvement. Participants also requested more information about their RDs, more patient-focused disease-specific RD organizations and seminars, and better awareness/education for the general public about RDs.	**Barrier** “Our biggest barrier has been medical professionals not understanding/knowing just how life threatening this disorder is and how imperative it is to treat it in an emergency at the very beginning of an emergency/crisis” (parent of a child with congenital adrenal hyperplasia) **Facilitator/Improvement** “Access to high quality medical information online so I could thoughtfully prepare for discussions with medical professionals” (woman in her 30s with non-radiographic axial spondyloarthritis) “Participation in the national foundation. They have advocate for legislation regarding our condition, the compile databases of recommended and knowledgeable healthcare providers, they hold a family conference to share the latest research, knowledge and treatment/care suggestions. This empowers me to be able to return to a less than knowledgeable doctor and be able to advocate for myself as help educate them. They also have established community that is able to share their experiences, symptoms, and suggestions to support each other.” (woman in her 30s with ectodermal dysplasia and parent to a child with RD) “I just wish more people knew about this condition. Having to define and explain it to healthcare providers who haven’t heard of it over and over is exhausting, and often they don’t know how to help because of their lack of knowledge about it. Giving explanations and asking questions that are often replied to with, “Gee, I don’t know. That sounds tough, good luck with that,” is extremely disheartening.” (woman in her 40s with idiopathic hypersomnia)
**Stigma, bias, discrimination, and self-doubt/** **Empowerment and advocacy**	**Barrier** Some participants shared experiences of stigma and discrimination when interacting with healthcare providers, such as when providers did not seem to consider their RD symptoms because they had other mental health or weight issues. These negative interactions caused stress, anger, anxiety, and self-stigma (e.g., self-loathing) that made participants hesitant to seek healthcare. **Facilitator/Improvement ** Some participants identified their personal sense of perseverance, persistence, self-advocacy, outlook, and positive attitude as facilitators. When participants were able to successfully navigate healthcare systems and connect with others with RDs, they reported feeling empowered.	**Barrier** “Many doctors don’t believe me when I report symptoms. I have learned to down play them (pain level for instance) for if I am honest all of a sudden I’m treated like a hysterical female and the doc stops listening. It’s not right and won’t help me in the long run or a doctor treating me, but I feel in order to get any care I must walk this line between honesty and keeping the doctor engaged. It has shown up in my medical record “she is not really sick” and “has a litany of complaints as usual” (woman in her 60s with cat scratch disease, babesiosis, and antiphospholipid syndrome) “Being misunderstood or people not UNDERSTANDING that they can’t understand. Solely, people just not understanding what my diagnosis actually means. It is so hard to suffer every day & have no one understand how hard it is to simply be awake. How hard I work every day to do the impossible & then belittle myself whenever I cannot make it possible. I am trying so hard to push myself past my limitations in hope I will succeed in the things that I will only ever reach in my dreams. While this internal battle within yourself is occurring, those around you only see “laziness” or “failure” on the surface.” (woman in her 20s with idiopathic hypersomnia) “Doctors would just tell me to lose weight and not do any tests to figure out what was wrong” (woman in her 70s with mixed connective tissue disease) “The second biggest barrier is my gender, I truthfully feel that because I am a black woman my needs are brushed aside. I get constantly told how strong I am, but I am not.” (woman in her 20s with hereditary amyloidosis) **Facilitator/Improvement** “Myself. I pushed for years for answers when doctor after doctor told me they couldn’t find anything seriously wrong with me. It took fully leaving the work force and putting my full time in finding answers to finally get a diagnosis.” (man in his 30s with hypophosphatasia) “A representative from my first co-pay program. Life had been pretty tough for me for several years, with misdiagnosis, loss of my job, loss of my house and initial denial from Social Security. She called me out of the blue, introduced herself and said “We are here to help.” To this day, it makes me cry. I tried to tell her in a letter how much those words meant to me. I couldn’t believe someone was contacting me to say they would help me. I had fought on my own for years. She was an angel.” (woman in her 50s with DYT-TOR1A) “A change in the culture of medicine, which currently deemphasizes patient experiences (symptoms, feelings, past medical experiences)and priorities visible-structural issues. This is particularly a problem for women and racial minorities, who are treated as attention seekers or hypochondriacs. Mutual respect and trust between medical professionals and their patients would improve communication, reduce negative experiences, and improve the quality of care and accuracy of diagnoses.” (woman in her 30s with narcolepsy)
**Nothing**	Many participants did not identify barriers, facilitators, or changes they hoped to see to improve healthcare access.	“Nothing” “Not applicable” “None” “Don’t know”

## Data Availability

The data presented in this article are not readily available due to their sensitive nature; participant responses may contain details that could identify them. Requests to access datasets should be directed to arhemmesch@stcloudstate.edu.
